# Computational Simulations of Thrombolytic Therapy in Acute Ischaemic Stroke

**DOI:** 10.1038/s41598-018-34082-7

**Published:** 2018-10-25

**Authors:** Andris Piebalgs, Boram Gu, Dylan Roi, Kyriakos Lobotesis, Simon Thom, Xiao Yun Xu

**Affiliations:** 1 Faculty of Engineering, Department of Chemical Engineering, South Kensington Campus, Imperial College London, London, SW7 2AZ United Kingdom; 2 Imaging Department, Charing Cross Hospital, Imperial College Healthcare NHS Trust, London, W6 8RF United Kingdom; 3 Faculty of Medicine, National Heart & Lung Institute, Hammersmith Campus, Imperial College London, London, W12 0NN United Kingdom

## Abstract

Ischaemic stroke can occur when an artery to the brain is blocked by a blood clot. The use of thrombolytic agents, such as tissue plasminogen activator (tPA), to dissolve the occluding clot is limited by the risk of intracerebral haemorrhage (ICH), a known side effect associated with tPA. We developed a computational thrombolysis model for a 3D patient-specific artery coupled with a compartmental model for temporal concentrations of tPA and lysis proteins during intravenous infusion of tPA, in order to evaluate the effects of tPA dose on the efficacy of thrombolytic therapy and the risk of ICH. The model was applied to a 3-mm-long fibrin clot with two different fibrin fibre radii in the middle cerebral artery (MCA) – a setting relevant to ischaemic stroke, and results for different tPA dose levels and fibrin fibre radii were compared. Our simulation results showed that clot lysis was accelerated at higher tPA doses at the expense of a substantial increase in the risk of ICH. It was also found that a fine clot with a smaller fibre radius dissolved much slowly than a coarse clot due to a slower tPA penetration into the clots.

## Introduction

Ischaemic stroke is one of the main causes of death and disability in the world. It occurs when blood clots occlude a cerebral artery thereby cutting off blood supply to a part of the brain. Ischaemic stroke can be treated with thrombolytic therapy that involves intravenous infusion of alteplase (recombinant tPA) to dissolve the occluding blood clot^1^. However, the treatment can cause severe side effects such as intracerebral haemorrhage (ICH), thereby limiting its application to a small subset of individuals^2^. Furthermore, since the occluding clots could be present at different locations in the cerebral vasculature with varying compositions, thrombolytic therapy can be ineffective in certain scenarios. Recently, there is new evidence suggesting that mechanical thrombectomy can be performed in combination with thrombolysis for best treatment outcomes^3,4^.

The efficacy of thrombolytic therapy depends on many factors; these include the location, size and composition of the clot, blood flow regime around the clot and drug dose. Owing to the short half-life of tPA (~4 minutes)^5^, continuous administration is required to maintain a sufficiently high concentration for clot dissolution. Currently, thrombolytic therapy involves intravenous infusion of tPA at 0.9 mg/kg with 10% given as a 1 minute bolus and the remaining 90% given over a continuous 1 hour infusion. This was the procedure adopted in the NINDS clinical trial^6^ which led to the FDA approval and wide-spread adoption of thrombolytic therapy for ischaemic stroke. However, other relevant clinical trials have used different tPA doses. For instance, the ECASS trial^7^ used a higher infusion dose of 1.1 mg/kg, while doses as low as 0.3 mg/kg have also been tested^8^. The J-MARS study^9^ evaluated the use of 0.6 mg/kg intravenous dose for the treatment of ischaemic stroke and found low levels of ICH (<5%) and relatively high levels of functional outcome at 3 months (>30%). Therefore, the optimal tPA dosing regimen for the treatment of ischaemic stroke remains contested and should be investigated to determine its effect on the efficacy of thrombolytic therapy.

Thrombolysis is a complex process that involves the interplays among multiple physical and biochemical phenomena, such as protein kinetics, blood flow and drug transport. Mathematical and computational models of clot lysis can be used to gain insights into the effect of individual factors on the efficacy of thrombolytic therapy. Considerable efforts have been made on the modelling of thrombolysis^10–16^, but existing mathematical models are confined to idealised geometries. Modelling the dissolution of clots in realistic settings is very challenging and computationally intense, and to the authors’ knowledge, there is no computational study so far that has investigated thrombolysis in patient-specific geometries. Furthermore, it is important to monitor the level of thrombolytic proteins in the plasma during tPA infusion for a reliable prediction of thrombolysis. Several studies employed a compartmental model for tPA and validated it against experimental data^17–19^. Although the model of Godfrey *et al*.^17^ used three compartments to describe the pharmacokinetics of tPA, reactions in the plasma were not included unlike the models of Noe & Bell^18^ and Tiefenbrunn *et al*.^19^.

In this study, we present a computationally efficient model that can evaluate clot lysis patterns for a given therapeutic regimen in a patient-specific geometry reconstructed from CT scans. This is achieved by coupling a 3D thrombolysis model with a compartmental model that describes the temporal evolution of lysis proteins in plasma during intravenous infusion of alteplase. The compartmental model and its parameters used in this study are taken from the work of Noe and Bell^18^ and Tiefenbrunn *et al*.^19^, so that both anti-plasmin and fibrinogen can be accounted for in addition to thrombolytic proteins. The lysis of a 3-mm-long fully occluding clot in the M1 segment of the middle cerebral artery (MCA) of an 80 kg man is used as an example to evaluate the effect of tPA dose on the efficacy of thrombolytic therapy. Three dose levels are simulated: low (0.6 mg/kg), normal (0.9 mg/kg) and high (1.2 mg/kg). The effectiveness of the treatment is determined by the breakthrough time and lysis completion time, while the risk of ICH is monitored according to the plasma concentration of fibrinogen. The latter is based on findings that low levels of fibrinogen concentration in plasma are associated with increased risk of ICH^20,21^.

## Modelling and Simulation Methods

**Figure 1:**
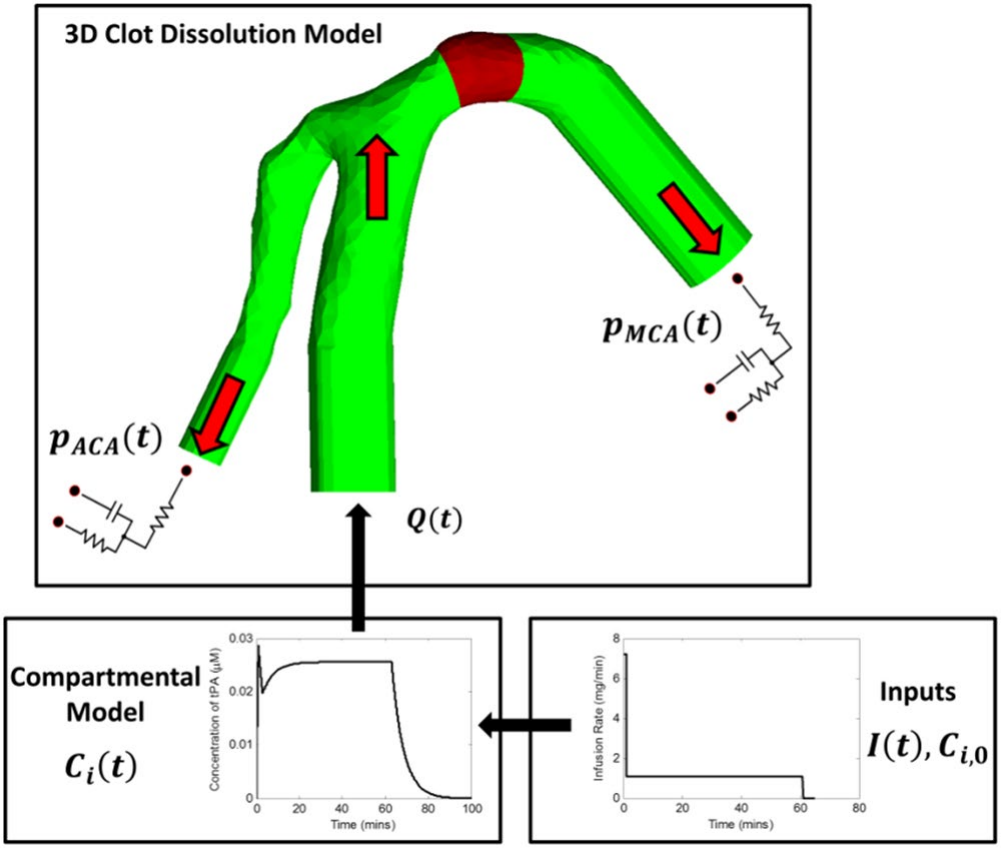
Overview of the computational model. The reconstructed arterial geometry represents the bifurcation of the internal carotid artery (ICA) into the anterior cerebral artery (ACA, left branch) and the MCA (right branch). Compartmental model solves transient plasma concentrations of free phase proteins for a given therapeutic regimen which are then used as inputs for the 3D clot dissolution model.

The computational model developed and used in this study includes: (i) a compartmental model to simulate the dynamic interactions of lysis proteins in plasma during intravenous infusion of tPA, and (ii) a 3D thrombolysis model that couples blood flow, drug transport and the dissolution of fibrin fibres. An overview of the integrated model is shown in Fig. 1, and details of the models are described below.

### Compartmental Model

A compartmental model was built to evaluate the temporal variations of tPA, plasminogen (PLG), plasmin (PLS), fibrinogen (Fbg) and antiplasmin (AP) in plasma during intravenous infusion of tPA. PLG is mainly consumed through its activation by tPA, a reaction that results in the production of PLS that then cleaves the fibrinogen molecules in plasma. PLS has a very short half-life and is quickly inhibited by AP. The role of plasminogen activator inhibitor, PAI-1, is neglected as its low concentration in plasma (~0.0003 µM) has a minor effect on changing the pharmacological level of tPA during thrombolytic therapy^22^. PLG, AP and fibrinogen (Fbg) are present at high levels in the plasma (2.2 µM, 1 µM and 8 µM, respectively) and are continuously generated. This system can be described by Equations (1–5).

1 $$\frac{d{C}_{tPA}}{dt}=-\,{k}_{HC}{C}_{tPA}+\frac{I(t)}{{M}_{w,tPA}{V}_{plasma}}$$

2 $$\frac{d{C}_{PLG}}{dt}=-\,{k}_{HC,PLG}{C}_{PLG}-\frac{{k}_{2,f}{C}_{tPA}{C}_{PLG}}{{K}_{M,f}+{C}_{PLG}}+{G}_{PLG}$$

3 $$\frac{d{C}_{PLS}}{dt}=-\,{k}_{HC,PLS}{C}_{PLS}+\frac{{k}_{2,f}{C}_{tPA}{C}_{PLG}}{{K}_{M,f}+{C}_{PLG}}-{k}_{AP}{C}_{AP}{C}_{PLS}$$

4 $$\frac{d{C}_{Fbg}}{dt}=-\,{k}_{HC,Fbg}{C}_{Fbg}-{k}_{cat,f}{C}_{Fbg}{C}_{PLS}+{G}_{Fbg}$$

5 $$\frac{d{C}_{AP}}{dt}=-\,{k}_{HC,AP}{C}_{AP}-{k}_{AP}{C}_{PLS}{C}_{AP}+{G}_{AP}$$

where *C*_*i*_ is the concentration of *i*-species (*i* = tPA, PLG, PLS, Fbg and AP), *V*_*plasma*_ the volume of plasma circulating in the human body, *M*_*w,tPA*_ the molecular weight of tPA, *k*_*HC,i*_ the rate of hepatic clearance of *i*-species, *k*_*2,f*_ and *K*_*M,f*_ the Michaelis-Menten coefficients relating to the rate of PLG activation in the free phase, *k*_*AP*_ the reaction constant for AP inhibition, *k*_*cat,f*_ the reaction constant for Fbg degradation, *G*_*i*_ the rate of generation of *i*-species in the bloodstream and *I*(*t*) the rate of infusion of tPA into the system as described by Equation (6) according to the conventional treatment protocol (10% in bolus and the remaining 90% in continuous infusion).

6 $$I(t)=\{\begin{array}{c}0.1\frac{{\alpha }_{tPA}w}{{t}_{B}},\,{\rm{i}}{\rm{f}}\,t\ge {t}_{0}\,{\rm{a}}{\rm{n}}{\rm{d}}\,t < {t}_{0}+{t}_{B}\\ 0.9\frac{{\alpha }_{tPA}w}{{t}_{C}},\,{\rm{i}}{\rm{f}}\,t\ge {t}_{0}+{t}_{B}+{t}_{D}\,{\rm{a}}{\rm{n}}{\rm{d}}\,t < {t}_{0}+{t}_{B}+{t}_{D}+{t}_{C}\\ 0,\,\,\,\,{\rm{o}}{\rm{t}}{\rm{h}}{\rm{e}}{\rm{r}}{\rm{w}}{\rm{i}}{\rm{s}}{\rm{e}}\end{array}$$

where *w* is the weight of the patient, *α*_*tPA*_ is the tPA dose (in mg/kg bodyweight), *t*_0_ refers to the time at the start of drug administration, *t*_*B*_ is the duration of bolus injection, *t*_*D*_ is the time delay between the bolus and continuous infusion and *t*_*C*_ is the duration of continuous infusion.

The hepatic clearance coefficient *k*_*HC,i*_ can be calculated using the half-life of *i*-species (*t*_*1/*2*,i*_) as given in Equation (7). The rate of generation of each species can be determined by assuming that it is counterbalanced by the rate of hepatic clearance at equilibrium. Assuming that the rate of generation is constant over time, it can be calculated using Equation (8) where *C*_*i,0*_ is the initial concentration of *i-*species. The values for all the kinetic constants can be found in Table 1.

7 $${k}_{HC,i}=\frac{\mathrm{ln}\,2}{{t}_{1/2,i}}$$

8 $${G}_{i}={k}_{HC,i}{C}_{i,0}$$

**Table 1:** Kinetic constants in the compartmental model.

Symbol	Value	Units	Source
*C*_*AP,0*_	1	µM	^44^
*C*_*Fbg,0*_	8	µM	^10, 18, 45^
*C*_*PLG,0*_	2.0	µM	^10, 46^
*C*_*PLS,0*_	0	µM	^10^
*C*_*tPA,0*_	0.07 × 10^−3^	µM	^47^
*k*_*2,f*_	0.3	1/s	^19^
*k*_*AP*_	10	1/(µM∙s)	^10^
*k*_*cat,f*_	6	1/(µM∙s)	Derived from^18^
*K*_*M,f*_	28	µM	^19^
*t*_*1/2,AP*_	2.64	days	^48^
*t*_*1/2,FBG*_	4.14	days	^45^
*t*_*1/2,PLG*_	2.2	days	^46^
*t*_*1/2,PLS*_	0.1	s	^49^
*t*_*1/2,tPA*_	4	mins	^5, 18^
*V*_*plasma*_	3.9	L	Max. volume for 80 kg patient^45^
*M*_*w,tPA*_	59.04	mg/µmol	—

The system of ordinary differential equations is solved with the 4^th^ order Runge-Kutta method using MATLAB R2017a. It requires specification of the drug infusion regimen and initial protein concentrations in the plasma. Solutions of the compartmental model provide temporal variations in the concentrations of free phase lysis proteins which are then used as inlet conditions for the 3D thrombolysis model (see Fig. 1).

### 3D Model of Thrombolysis

A 3D model of the ICA bifurcation is reconstructed from CT images obtained from a patient as part of a standard examination by means of the segmentation software Materialise Mimics 19.0. The images were anonymised before processing, and no ethics approval is required for the use of these images. The reconstructed ICA bifurcation consists of the M1 segment of the MCA and the A1 segment of the ACA (Fig. 1). A 3-mm-long clot is artificially placed in the M1 segment of the MCA to simulate an M1 occlusion which is commonly observed in clinical studies^23^. In our previous work^24^, fibrinolysis was modelled as a continuous shrinking of a homogeneous fibrin fibre radius depending on the bound plasmin concentration, and the time-varying fibrin fibre radius was then used to evaluate changes in clot permeability and its resistance to fluid flow. This is modified by considering the degradation of fibrin binding sites in the current model, whereby fibrinolysis is described as a reduction of the fibrin network. The mathematical descriptions of fibrinolysis are presented below, while details on modelling blood flow in the presence of blood clot can be found in Appendix A.

#### Transport of Free Phase Proteins

The transport of free phase proteins is described by a set of convection-diffusion-reaction equations.

9 $$\frac{\partial {C}_{i}}{\partial t}+{\boldsymbol{u}}\nabla {C}_{i}=\nabla [{D}_{i}\nabla ({C}_{i})]-{\rm{\Sigma }}{R}_{i}$$

where *C*_*i*_ is the concentration of the free phase *i*-species and *D*_*i*_ is the diffusion coefficient of *i*. The last term ${\rm{\Sigma }}{R}_{i}$ corresponds to the net consumption of *i* and is determined in part by the net adsorption and desorption of the species in the fibrin network for tPA, PLG and PLS:

10 $${\rm{\Sigma }}{R}_{i}={k}_{a,i}{C}_{i}{n}_{free}-{k}_{r,i}{n}_{i}$$

where *k*_*a,i*_ and *k*_*r,i*_ are the adsorption and desorption coefficient of *i*, respectively, *n*_*free*_ is the concentration of free binding sites and *n*_*i*_ is the concentration of bound *i* species. Additional terms for PLS are included to account for inhibition by AP and solubilisation of species following dissolution.

11 $${\rm{\Sigma }}{R}_{PLS}={k}_{AP}{C}_{AP}{C}_{PLS}-{k}_{r,PLS}{L}_{PLS}$$

12 $${\rm{\Sigma }}{R}_{AP}={k}_{AP}{C}_{AP}{C}_{PLS}$$

where *k*_*AP*_ is the reaction rate coefficient of the inhibition of PLS by AP and *L*_*PLS*_ is the concentration of fibrin with PLS still attached to it. By introducing the second term *k*_*r,PLS*_*L*_*PLS*_ in Equation (11), the amount of PLS that returns to the free phase can be taken into account.

#### Fibrinolytic Reactions of Bound Proteins

The equations that describe the bound species are based on the kinetics of fibrinolysis. Bound PLG and bound tPA react together to produce bound PLS via a Michaelis-Menten reaction. The protease then goes on to dissolve the binding sites on the fibrin fibre. This set of equations is written as:

13 $$\frac{\partial {n}_{tPA}}{\partial t}={k}_{a,tPA}{C}_{tPA}{n}_{free}-{k}_{r,tPA}{n}_{tPA}$$

14 $$\frac{\partial {n}_{PLG}}{\partial t}={k}_{a,PLG}{C}_{PLG}{n}_{free}-{k}_{r,PLG}{n}_{PLG}-\frac{{k}_{2}{n}_{PLG}{n}_{tPA}}{{K}_{M}+{n}_{PLG}}$$

15 $$\frac{\partial {n}_{PLS}}{\partial t}={k}_{a,PLS}{C}_{PLS}{n}_{free}-{k}_{r,PLS}{n}_{PLS}+\frac{{k}_{2}{n}_{PLG}{n}_{tPA}}{{K}_{M}+{n}_{PLG}}-{k}_{cat}\gamma {n}_{PLS}$$

16 $$\frac{\partial {L}_{PLS}}{\partial t}={k}_{cat}\gamma {n}_{PLS}-{k}_{r,PLS}{L}_{PLS}$$

17 $$\frac{\partial {n}_{tot}}{\partial t}=-\,{k}_{cat}\gamma {n}_{PLS}$$

18 $${n}_{free}={n}_{tot}-({n}_{tPA}+{n}_{PLG}+{n}_{PLS})$$

where *n*_*tot*_ is the total concentration of binding sites, *k*_*cat*_ is the reaction coefficient for the degradation of fibrin by PLS, *γ* is the solubilisation constant and *k*_2_ and *K*_*M*_ are the Michaelis-Menten coefficients of PLG activation.

Changes in permeability of the clot are evaluated based on the total number of binding sites in the system.

19 $${\varphi }_{f}={\varphi }_{f,0}(1-{E}_{L})$$

20 $${E}_{L}=1-\frac{{n}_{tot}}{{n}_{tot,0}}$$

21 $$k=\{\begin{array}{cc}\frac{\beta {R}_{f0}^{2}}{16{\varphi }_{f}^{1.5}(1+56{\varphi }_{f}^{3})} & {\rm{w}}{\rm{h}}{\rm{e}}{\rm{n}}\,{E}_{L} < {E}_{L,crit}\\ {\rm{\infty }} & {\rm{w}}{\rm{h}}{\rm{e}}{\rm{n}}\,{E}_{L}\ge {E}_{L,crit}\end{array}$$

where *ϕ*_*f*_ is the volume fraction of fibrin, *ϕ*_*f,0*_ the initial volume fraction, *E*_*L*_ is the extent of lysis, *n*_*tot,0*_ the initial concentration of binding sites, *k* the clot permeability^25^, *β* is the permeability coefficient. *E*_*L,crit*_ is the critical extent of lysis, which is used to define a region that is sufficiently lysed to restore local blood flow, and its value is assumed to be 0.95 (instead of 1 for reduced computational cost) based on previous experimental study^26^. The initial number of binding sites and the initial fibrin volume fraction can be determined by considering the average fibrin fibre radius and initial fibrin density^27^ and further details can be found in Appendix A. The coefficients needed for the solution of the model described above are listed in Table 2.

**Table 2:** Kinetic and transport parameters used in the simulation.

Symbol	Description	Value	Units	Source
*D*_*α*_	Dispersion coefficient for tPA, PLG and PLS	5 × 10^−11^	m^2^/s	^50^
*k*_*2*_	Michaelis reaction rate coefficient	0.3	1/s	^51^
*k*_*a,PLG*_	Adsorption coefficient for PLG	0.1	1/(μM·s)	^52^
*k*_*a,PLS*_	Adsorption coefficient for PLS	0.1	1/(μM·s)	^52^
*k*_*a,tPA*_	Adsorption coefficient for tPA	0.01	1/(μM·s)	^53^
*k*_*cat*_	Lysis coefficient	2.178	1/s	^54^
*K*_*M*_	Michaelis constant	0.16	μM	^55^
*k*_*r,PLG*_	Desorption coefficient for PLG	3.8	1/s	^56^
*k*_*r,PLS*_	Desorption coefficient for PLS	0.05	1/s	^52^
*k*_*r,tPA*_	Desorption coefficient for tPA	0.0058	1/s	^53^
*β*	Permeability Coefficient	0.286		Derived from^25^
*E*_*L,crit*_	Critical extent of lysis	0.95	—	^26^
1/*γ*	Cuts needed for PLS to cut 1 fibrin unit	10	—	^52^
*μ*	Viscosity of blood	3.5 × 10^−3^	m^2^/s	^57^

#### Implementation of Model Equations and Simulation Details

The equations governing the fluid flow, transport of lysis proteins and fibrinolytic kinetics are coded in the C++ programming language and implemented in OpenFOAM 4.0, an open source computational fluid dynamics (CFD) code. The reconstructed ICA bifurcation is imported into ICEM CFD 15.0 to generate a structured mesh consisting of around 0.9 million elements, which is sufficient to achieve grid independence for predicted lysis time (approximately 10 seconds difference between meshes of 0.9 million and 1.6 million). The PIMPLE algorithm available in OpenFOAM 4.0 is used for pressure-velocity coupling, where a tolerance of 10^−5^ and 5 × 10^−5^ for velocity and pressure are chosen to ensure efficient convergence and sufficient accuracy in each time step of 0.01 second.

A steady flowrate of 4.31 mL/s, calculated by averaging the pulsatile ICA flow waveform in the literature^28^, is specified at the inlet. At the MCA and ACA outlets, physiological pressure outflow boundary conditions are defined using a 3-element Windkessel model^29^, whose equations and parameters are included in Appendix A of the Supporting Information. Arterial walls are assumed to be rigid and no-slip. The initial values of pressure and velocity are set to 0 for the entire computational domain. At the ICA inlet, temporal variations of each free species at every time-step are specified based on results obtained from the compartmental model.

## Results

A total of 6 simulations are carried out for three tPA doses (0.6 mg/kg, 0.9 mg/kg and 1.2 mg/kg) and two fibrin fibre radii (30 nm and 100 nm to represent a fine and coarse clot, respectively). A bolus of 10% of the total tPA dose is given initially within 1 minute and the remaining 90% is administered during a 1-hour continuous infusion. A delay of 1 minute is arbitrarily chosen to simulate a quick transition from bolus to continuous infusion. The average fibre radii chosen for simulations are based on experimental observations in the literature^30,31^.

### Infusion of Alteplase during Ischaemic Stroke

**Figure 2:**
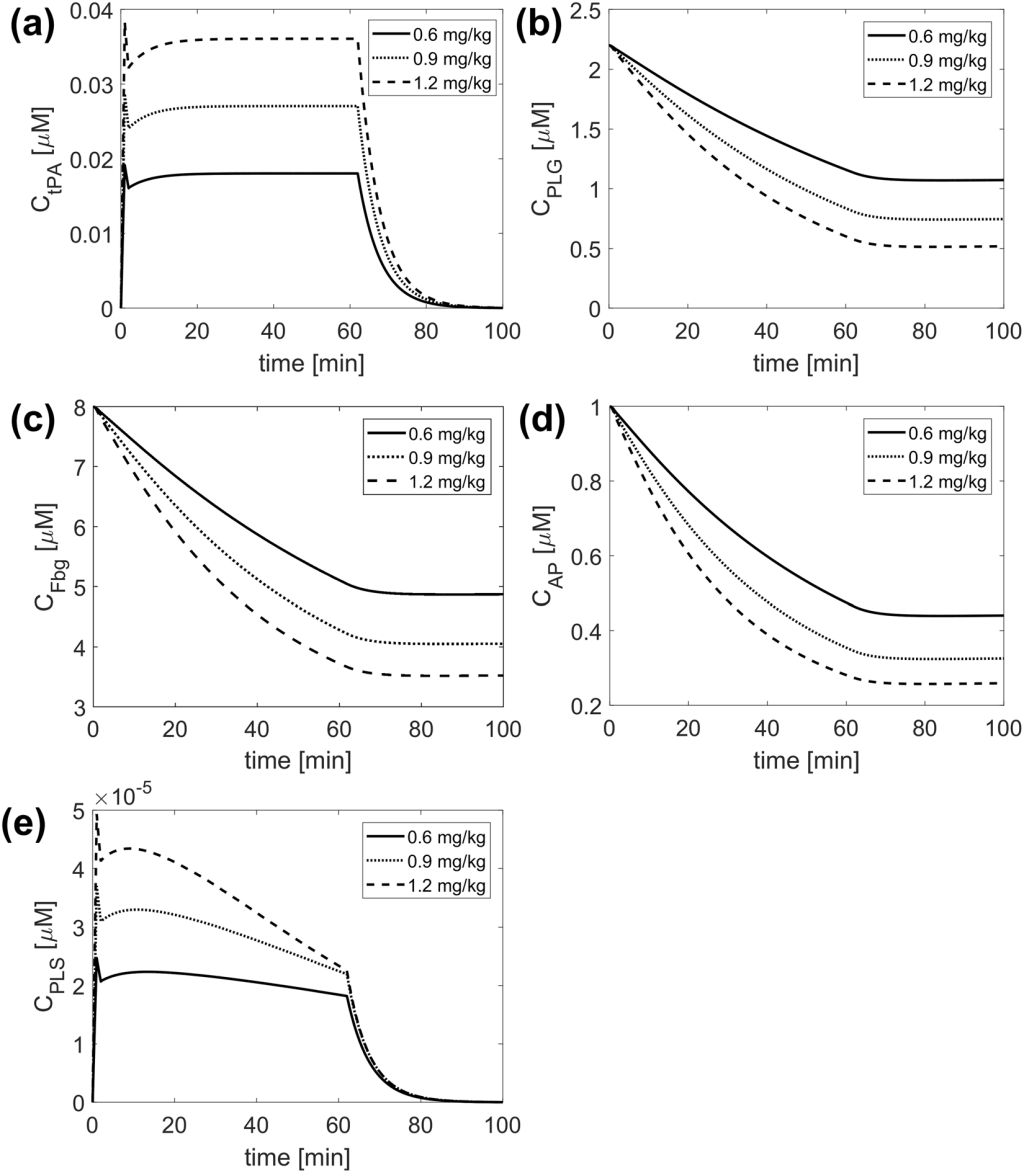
Results obtained from the compartmental model for free phase concentrations of (**a**) tPA (**b**) PLG (**c**) fibrinogen (**d**) AP and (**e**) PLS for different tPA doses: 0.6 mg/kg (solid), 0.9 mg/kg (dotted line) and 1.2 mg/kg (dashed line).

First, the compartmental model is validated against experimental data in the literature^5,18,32^ and detailed comparisons can be found in Appendix B. Using the kinetic constants and initial values given in Table 1, changes in plasma concentrations of PLG, tPA, AP, PLS and Fbg are determined for different tPA doses. Results obtained from the compartmental model for tPA, PLG, Fbg, AP and PLS are shown in Fig. 2. Since the compartmental model simulates a well-mixed system, it only provides temporal variations of the modelled species which are used to prescribe free phase protein concentrations at the inlet of the 3D clot dissolution model.

It can be seen that the bolus allows for a rapid increase in the free phase concentration and the continuous infusion allows for a steady tPA concentration to be sustained (Fig. 2(a)). The delay between the bolus and continuous infusion causes a sharp drop in free phase tPA concentration, which is more noticeable at higher tPA doses. Increasing the tPA dose causes more rapid reductions in PLG (Fig. 2(b)) and fibrinogen levels (Fig. 2(c)), and for all 3 tPA doses, the level of PLS in the circulation is very low (Fig. 2(e)) due to its rapid inhibition by AP (Fig. 2(d)).

### Blood Flow Patterns

**Figure 3:**
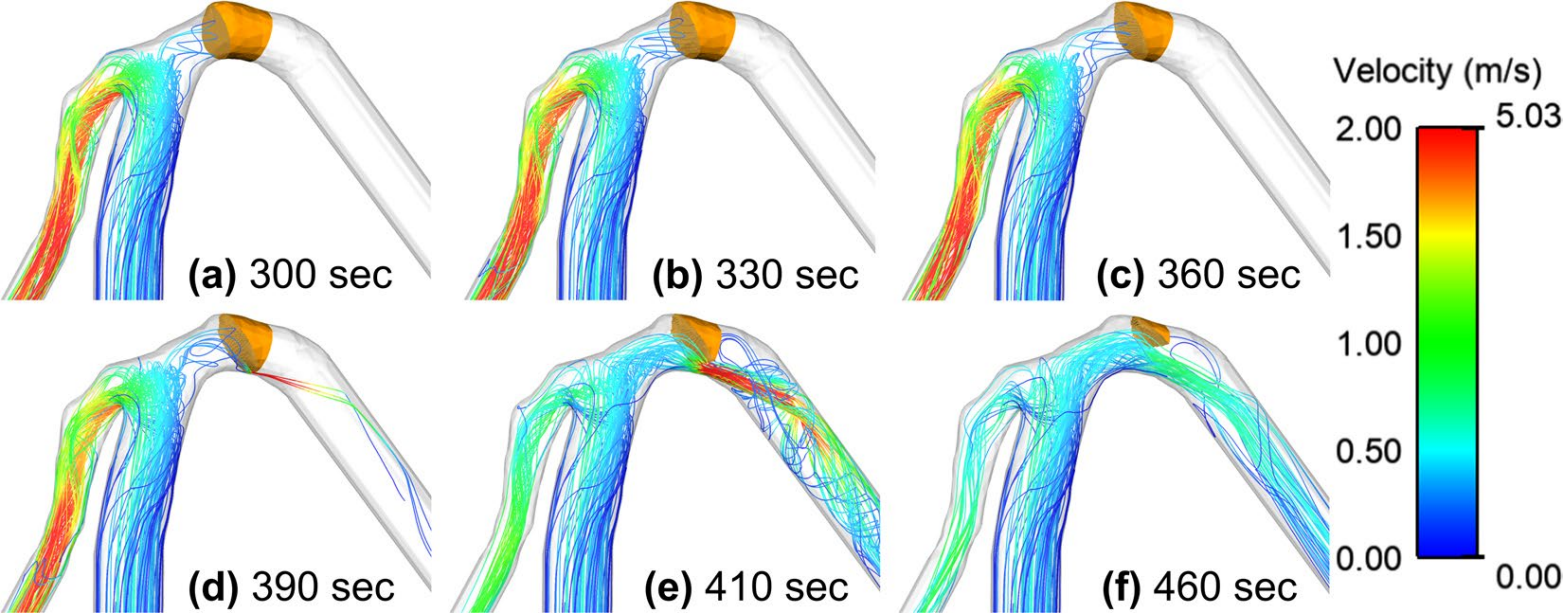
Velocity streamlines at different time points during the thrombolysis of the fine clot for a tPA dose of 0.9 mg/kg and a delay of 1 min. The remaining clot region is identified based on *E*_*L*_ < *E*_*L,crit*_. Colour corresponds to the magnitude of velocity in m/s. Left branch is the ACA and the right branch is the MCA.

Figure 3 shows the velocity streamlines throughout the thrombolytic process along with the remaining clot volume (region with *E*_*L*_ < *E*_*L,crit*_) for the fine clot with a tPA dose of 0.9 mg/kg. Initially, the blood clot occludes the MCA (a) and forces blood to flow into the ACA. As the thrombolytic agent is introduced into the system, the clot is gradually dissolved starting from the lower side along the inner curvature of the MCA (b, c). Further dissolution of the clot enables blood to pass through the clot in a form of high velocity jet (d). This asymmetric jet along the inner curvature of the MCA expands as lysis proceeds (e) until blood flow in the MCA is partially restored (f). The shape of the remaining clot is asymmetric with the top side of the clot being dissolved more slowly than its lower side. As a result, the dissolving clot is thicker at the top and thinner at the bottom. After breakthrough is achieved, the remnant mural clot is attached to the arterial wall along its outer curvature with an oblique lysis front facing the flow. Detailed lysis patterns and spatial distributions of key parameters are presented in a later section.

### Changes in Arterial Flowrate and Pressure

**Figure 4:**
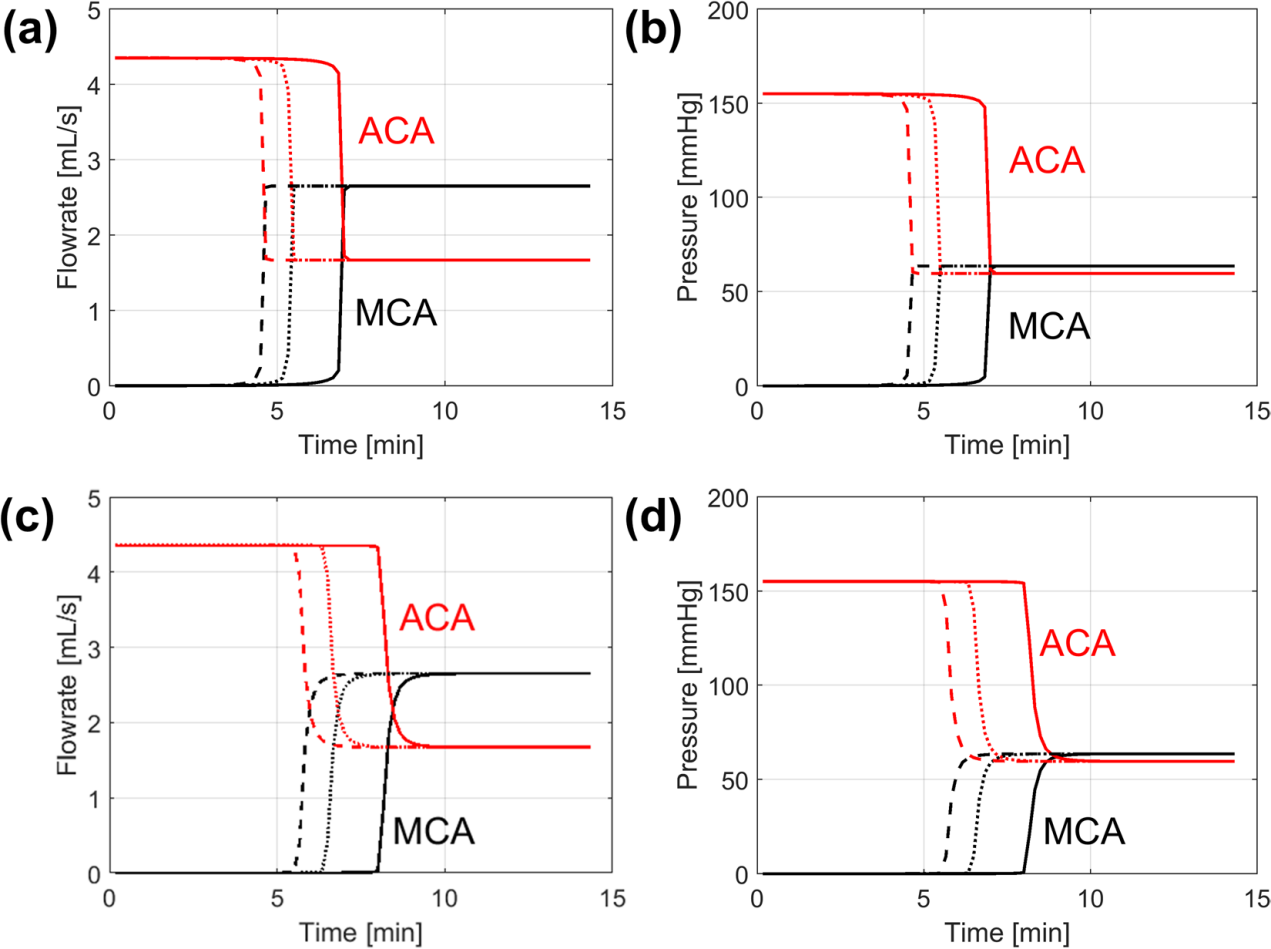
Blood flowrates and pressure in the MCA (black) and ACA (red) over time for tPA doses of 0.6 mg/kg (solid), 0.9 mg/kg (dotted) and 1.2 mg/kg (dashed) for the coarse clot (**a** and **b**) and the fine clot (**c** and **d**). (**a**) Flowrates and (**b**) pressures in ACA and MCA for the coarse clot (**c**) flowrates and (**d**) pressures in ACA and MCA for the fine clot.

Figure 4 shows the predicted changes in the ACA and MCA flowrate and pressure for the 3 different tPA doses for the coarse (a, b) and fine clots (c, d). For both clots, the ACA flowrate starts at an initially high value of around 4.3 mL/s which then drops to around 1.6 mL/s upon clot breakthrough. In a similar manner the MCA flowrate rises from 0 to around 2.7 mL/s following re-establishment of flow in the MCA branch. Changes in pressure at each outlet mirror changes in the corresponding flowrate. The pressure difference between the ACA and MCA outlets falls from an initial value of around 150 mmHg to 0 following clot dissolution.

These results also show the influence of tPA dose and fibrin radius on the breakthrough time. The predicted breakthrough times are approximately 5 to 7 minutes for the coarse clot and 7 to 10 minutes for the fine clot. After an abrupt change in flowrate and pressure following clot breakthrough, both haemodynamic variables remain steady.

### Changes in Clot Volume, Extent of Lysis and Free Phase tPA Concentration

**Figure 5:**
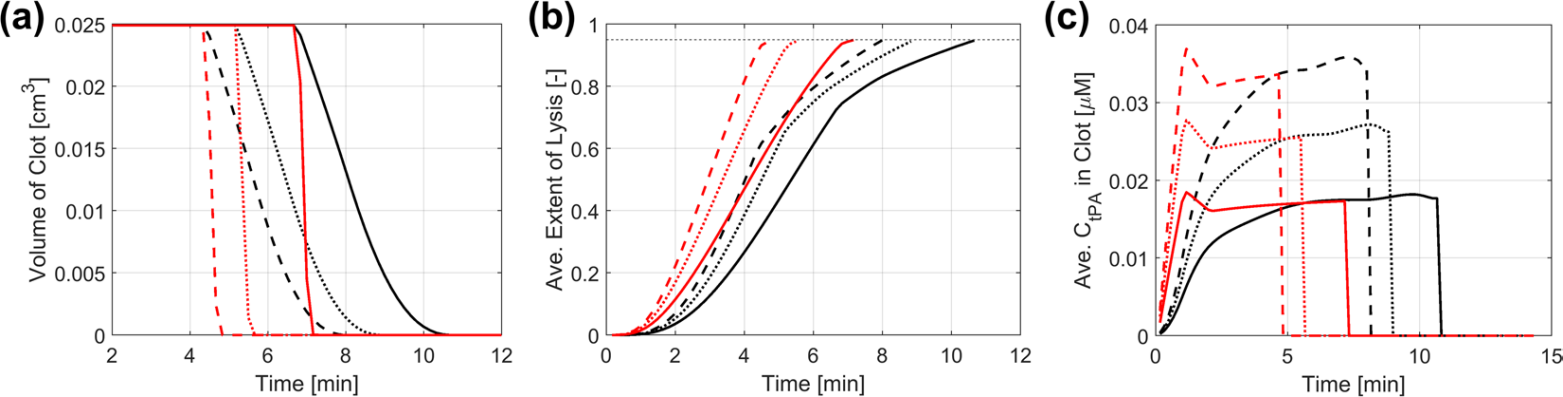
Changes in (**a**) Clot volume (**b**) extent of lysis and (**c**) free phase tPA concentration averaged over the remaining clot domain. Results are presented for the fine (black) and coarse (red) clots and different tPA doses of 0.6 mg/kg (solid), 0.9 mg/kg (dotted) and 1.2 mg/kg (dashed).

The rate of lysis for different tPA doses and fibrin radii can be assessed by monitoring the volume of the remaining clot as shown in Fig. 5(a). Starting with an initial volume of 0.025 cm^3^, reduction in clot volume begins at 4.3–6.7 mins and the clot diminishes at 4.8–10.7 mins depending on the tPA dose and clot type. It is seen that the coarse clot (red in Fig. 5(a)) dissolves much faster than the fine clot (black in Fig. 5(a)), although changes in clot volume start at a similar time for the same tPA dose. It is also found that for each clot the lysis rate is dependent on the amount of tPA injected and thrombolysis is accelerated at higher tPA doses, albeit not proportionally. Figure 5(b) shows the extent of lysis averaged over the remaining clot domain. Although the clot volume stays constant in the first few minutes (due to *E*_*L*_ < *E*_*L,crit*_), the extent of lysis increases gradually for both the fine and coarse clots.

Figure 5(c) compares variations of free phase tPA concentration within each clot averaged over the remaining clot volume. The initial bolus infusion results in a surge in the circulating concentration of tPA in the coarse clot, with a linear relationship between average tPA concentration and time during this phase. The fine clot, however, shows a much slower increase in the free phase tPA concentration due to its lower permeability and greater resistance, which will be elaborated in the following section.

### Spatial Distributions of Lysis Proteins and Extent of Lysis

**Figure 6:**
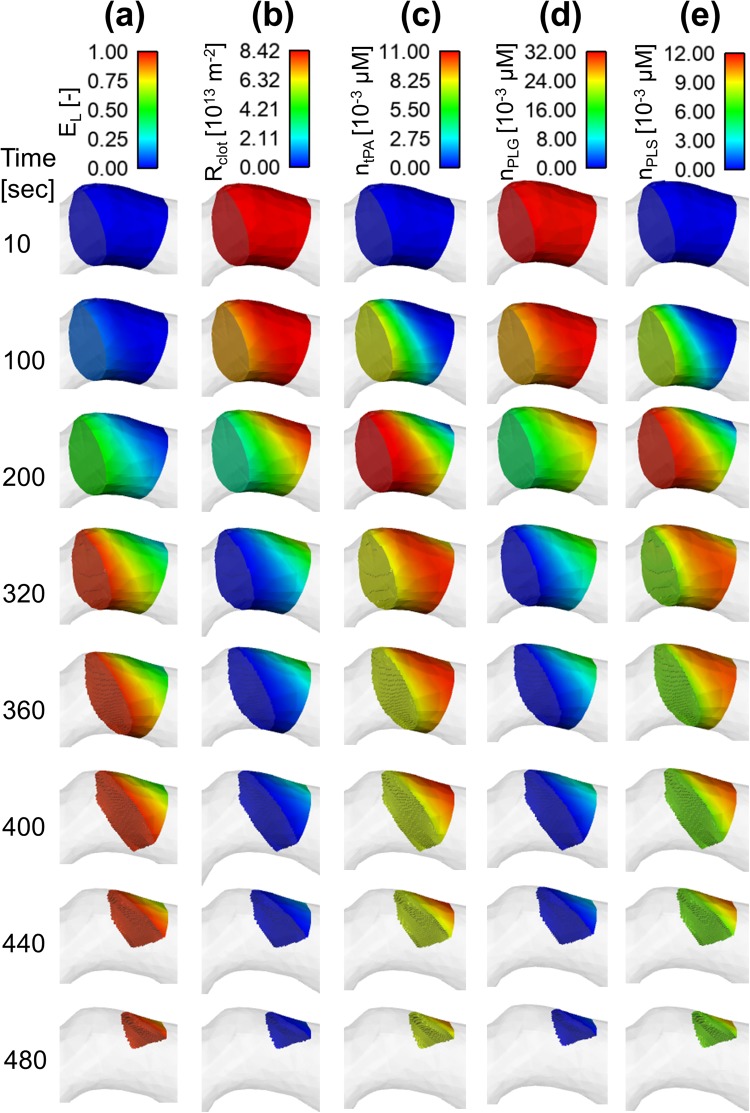
Spatial distributions for the fine clot (**a**) extent of lysis (*E*_*L*_) (**b**) hydraulic resistance of clot (*R*_*clot*_) (**c**) bound tPA concentration (*n*_*tPA*_) (**d**) bound plasminogen concentration (*n*_*PLG*_) and (**e**) bound plasmin concentration (*n*_*PLS*_).

**Figure 7:**
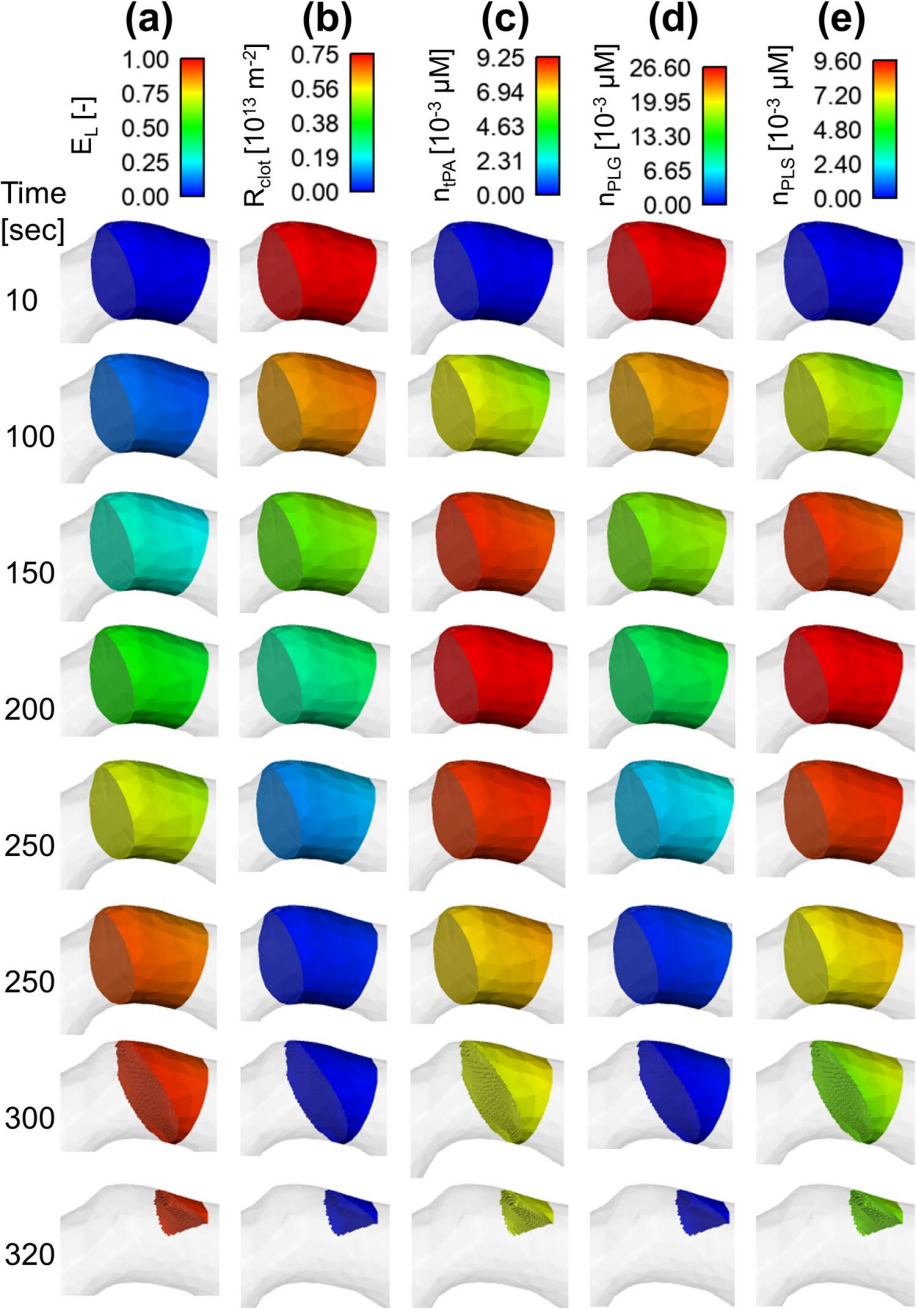
Spatial distributions for the coarse clot (**a**) extent of lysis (*E*_*L*_) (**b**) hydraulic resistance of clot (*R*_*clot*_) (**c**) bound tPA concentration (*n*_*tPA*_) (**d**) bound plasminogen concentration (*n*_*PLG*_) and (**e**) bound plasmin concentration (*n*_*PLS*_).

Spatial and temporal variations of key variables are presented in Figs 6 and 7 for a tPA dose of 0.9 mg/kg; these include the extent of lysis, hydraulic resistance of clot (defined as ${R}_{clot}=\frac{1}{k}$), and concentrations of bound phase proteins (tPA, PLG and PLS) at different time points for the fine and coarse clots, respectively.

By comparing results for the fine and coarse clots, it is clear that the fine clot exhibits considerable spatial variations in all the variables, but the coarse clot shows a fairly uniform distribution throughout. For example, the extent of lysis for the fine clot (Fig. 6(a)) increases gradually from the clot front to the rear over time towards the threshold value of 0.95. In contrast, the extent of lysis for the coarse clot (Fig. 7(a)) only varies with time and there is hardly any change along the clot length. This explains the unusual clot volume curve for the coarse clot observed in Fig. 5(a) where there is a sharp fall at the start of lysis, and the clot disappears in a very short time. Likewise, the clot resistance follows the same pattern, but in reverse order.

Interestingly, the concentration of bound tPA in the fine clot (Fig. 6(c)) peaks at the clot front at around 200 seconds and then starts to fall at the front with its peak moving towards the rear. This is because the rate of tPA desorption from the binding sites of the clot is faster than the rate of adsorption onto the binding sites when bound tPA concentration is high. The bound PLS concentration (Fig. 6(e)) shows a similar pattern to bound tPA, but the reduction in bound PLS concentration at the clot front over time is more likely attributed to the combined effect of accelerated degradation and desorption rates caused by high bound PLS concentration, as described by Equations (13–18). For bound PLG (Fig. 6(d)), its concentration is initially high due to the high concentration of free phase PLG in plasma.

## Discussions

In this study, the effects of tPA dose and fibrin radius of fibrin clots on the efficacy of thrombolytic therapy was examined through the use of a computational model. Changes in the concentration of lysis proteins over time during intravenous infusion of alteplase were simulated via a compartmental model. In order to ensure its accuracy, the model results were compared with experimental data (shown in Appendix B) and were found in good agreement with the measurements of Tanswell *et al*.^5^ for a continuous infusion of alteplase at 0.25 mg/kg and 0.5 mg/kg, and the experimental data of Collen *et al*.^32^ for a higher dose at 0.75 mg/kg over a 90 minute period.

Although continuous infusions were adopted in the experimental studies mentioned above, thrombolytic therapy for ischaemic stroke typically involves 2 modes of tPA administration: a bolus of 10% of the entire dose over 1 minute, followed by a continuous infusion of the remaining 90% administered over 60 minutes^6^. The dose can range from 0.6 mg/kg^9^ to 1.1 mg/kg^7^. Furthermore, a delay of up to 8 minutes can occur between the administration of the bolus and continuous infusion. Acheampong *et al*.^33^ found that although there was no difference in 3-month outcomes for delays longer than 8 minutes, there was a slight decrease in the likelihood of functional independence. In the simulations presented here, a modest 1 minute delay was applied, which caused a sharp drop in free phase tPA concentration as shown in Fig. 2(a). Smith *et al*.^34^ showed that increasing the delay in starting continuous infusion could significantly affect serum tPA levels, which might reduce the efficacy of thrombolysis.

An interesting observation from the compartmental model simulation results was the slow recovery of fibrinogen and PLG after the infusion of alteplase. Low fibrinogen levels have been implicated in the development of ICH, with concentrations around 150 mg/dL or lower being linked to an increased risk of bleeding^20^. In addition to this, a low concentration of PLG can be associated with suppressed lytic efficacy^35^. Thus, although a high tPA dose is expected to enhance the rate of thrombolysis, the increased risk of ICH (due to low fibrinogen levels) and reduction in lytic efficacy (caused by low PLG concentrations) would limit its use. For instance, a tPA dose of 1.2 mg/kg would reduce the fibrinogen level by 50% in the simulated case. Since the initial concentration of fibrinogen can vary between 5 and 13 µM^18^, a 50% reduction could cause the fibrinogen level to fall to a very low level for certain patients. As such, using high levels of tPA may be favourable for some patients whilst for others it might cause an unacceptable risk of ICH.

The compartmental model, when used in conjunction with our 3D model of clot dissolution, can predict clot lysis in a clinically relevant setting. The streamlines and change in clot shape (in Fig. 3) showed the development of an asymmetric lysis front that gave rise to a local increase in the blood velocity due to flow constriction. The formation of a sharp lysis front in a patient-specific geometry could be due to 2 reasons. Firstly, the presence of the arterial curvature allowed for the formation of a highly skewed velocity pattern that encouraged the transport of the drug into a specific section of the clot. Secondly, although the length of the clot along the centreline was 3 mm, the curvature of the MCA caused an initially inhomogeneous clot length (Fig. 3(a)). Since pressure exerts a uniform force normal to the surface of the clot, the perfusion velocity would be higher through the shorter clot side (close to the inner curvature of the arterial wall). This difference in perfusion velocity resulted in the formation of a non-uniform lysis front. It is interesting to note that the changing clot shape was not affected by the tPA concentration, so the same lysis front was maintained for all the tPA doses examined. The simulation results showed that the occurrence of a mural clot following thrombolysis was affected by the geometry of the artery, and this could be an indicator of future complications. Mural clots can cause re-stenosis if not treated properly and may lead to distal embolisms.

The model can also predict changes in the MCA and ACA flowrate and pressure (Fig. 4). The drastic change in ACA and MCA flowrates marked the moment when clot breakthrough occurred. Our simulations showed that the flowrates for the MCA and ACA after clot dissolution were 2.7 mL/s and 1.6 mL/s, respectively. These are slightly higher than the corresponding values (2.1 mL/s and 1.5 mL/s) reported in the literature^36,37^, which can be attributed to large individual variations of mean flow going into the ICA that can range from 3.4 mL/s to 5.4 mL/s^38^. The predicted changes in pressure at the MCA and ACA outlet mirrored the changes in flowrate in that the pressure across the clot, which can be roughly estimated as (*P*_ACA_ − *P*_MCA_), fell rapidly to 0 as clot breakthrough was achieved. The average pressure drop across the clot was around 150 mmHg before clot lysis, which is within the range expected for acute occlusions^39^.

Quantitative information on the degradation of clot over time can be obtained by examining the volume of clot and average extent of lysis shown in Fig. 5. Our simulations showed that it took between 4 to 10 minutes to achieve complete clot dissolution depending on the fibrin fibre radius in the clot and tPA dose. As expected, the rate of degradation with the highest tPA dose was noticeably faster than with the other two doses. However, the lysis times predicted by the simulations were much shorter than clinical observations (23 ± 16 minutes) made on 43 patients with their MCA or basilar artery occluded^1^. This discrepancy was most likely attributed to the short length of the clot and the assumption that the clot was composed of only fibrin fibres without any cellular components. It was reported that red blood cells and platelets were abundant in clots retrieved from acute stroke patients^40^, which can slow down fibrinolysis^40,41^.

Furthermore, spatial variations of clot resistance and concentrations of lysis proteins were compared between the fine and coarse clots. The fine fibrin clot had approximately 10 times higher initial resistance than the coarse clot, that is, the permeability of the fine clot was 10 times lower. As a consequence, tPA penetration through the fine clot was much slower than that within the coarse clot, and spatial distributions of lysis proteins were also different, with the coarse clot presenting a fairly uniform pattern (Fig. 7) whilst the fine clot displaying considerable variations along the clot length (Fig. 6). As mentioned before, intravenous infusion of alteplase is usually given in 2 stages, with a bolus intended to rapidly increase the initial tPA concentration. It can be seen that for a coarse fibrin clot, the average concentration of tPA in the clot (Fig. 5(c)) for different tPA doses mirrored the inlet conditions determined by the compartmental model. However, it is clear that the bolus injection had a less pronounced effect on a fine fibrin clot. In this case, the 1 minute bolus of tPA caused a moderate increase in the free phase tPA concentration, with the continuous infusion allowing for a gradually increased penetration until a steady state was reached. These results suggest that for a less permeable clot (as in a fine clot), a bolus injection may not be necessary and would only introduce a time delay until the continuous infusion is provided.

Given that the resistance of clot can differ by up to two orders of magnitude depending on clot compositions^30^, and that the initial heterogeneous nature of the clot is expected to affect the clot lysis pattern^42^, an optimal tPA dose should be determined according to the composition of the clot as well as the patient’s own fibrinogen levels. Since complete clot dissolution is also dependent on the movement of the lysis front within the clot, a fully occlusive clot with a low permeability and a long length would be less sensitive to the tPA dose. On the other hand, retracted clots which have a different structure with tessellated erythrocytes localised in the centre and network of fibrin fibres and platelets in the periphery^43^, may respond more strongly to the choice of tPA dose. The influence of different clot types with additional cellular components to the fibrin network on clot lysis pattern and dissolution time should be examined in future studies. Although our results have been compared and justified using data available in the literature, the lack of spatially resolved experimental data prevented us from corroborating the spatial patterns predicted by our 3D clot lysis model.

The current computational study involves several assumptions. First, laminar flow was assumed throughout the lysis process. Although the Reynolds number was around 290 based on the average MCA diameter and flowrate, a high velocity jet was present in the very early stage of clot breakthrough as seen in Fig. 3(d), which may cause the flow to become temporarily turbulent. Furthermore, the pulsatile nature of arterial flow was ignored and a steady flowrate at the ICA inlet was used. This assumption was made because of the different time scales of clot lysis and blood pulsation in arteries; the former is a much slower process compared to the cardiac cycle period which is around 1 s. Our preliminary assessment using an idealised geometry and accelerated lysis kinetics showed that the predicted lysis time and pattern were not affected by the steady flow assumption. In the future, we will perform physiologically realistic pulsatile flow simulations with real kinetics in patient-specific geometries in order to capture the instantaneous response of intra-arterial pressure and flowrate during the course of lysis.

## Electronic supplementary material

Supporting information

## Data Availability

The datasets generated during and/or analysed during the current study are available from the corresponding author on reasonable request.
