# Supplementary material for: Computational Simulations of Thrombolytic Therapy in Acute Ischaemic Stroke
Source: Sci Rep. 2018 Oct 25;8:15810. doi: 10.1038/s41598-018-34082-7 (PMC6202379; doi:10.1038/s41598-018-34082-7)
Supplement: Supplementary file 1 — Supporting information [file 41598_2018_34082_MOESM1_ESM.pdf]

Supporting Information

Computational Simulations of Thrombolytic Therapy in Acute Ischaemic Stroke

Andris Piebalgs[1*], Boram Gu[1*], Dylan Roi[2], Kyriakos Lobotesis[2], Simon Thom[3], Xiao Yun Xu[1,+]

[1]Faculty of Engineering, Department of Chemical Engineering, South Kensington Campus, Imperial College London, London SW7 2AZ, United Kingdom

[2]Imaging Department, Charing Cross Hospital, Imperial College Healthcare NHS Trust, London W6 8RF, United Kingdom

[3]Faculty of Medicine, National Heart & Lung Institute, Hammersmith Campus, Imperial College London, London W12 0NN, United Kingdom

+ Correspondence and requests for materials should be addressed to X.Y.X (yun.xu@imperial.ac.uk)

* These authors contributed equally to this work.

Appendix A. Computational Models

A.1 Fluid Flow

Fluid flow is solved using the modified Navier-Stokes equations derived from the conservation of mass and momentum. An additional momentum source term is included to account for the resistance of blood clot to fluid flow.

$$\nabla \cdot \boldsymbol{u} = 0 \qquad \text{Equation (A.1)}$$

$$\rho \left(\frac{\partial \boldsymbol{u}}{\partial t} + \boldsymbol{u} \cdot \nabla \boldsymbol{u} \right) = -\nabla p + \mu \nabla^2 \boldsymbol{u} - \frac{\mu}{k} \boldsymbol{u} \qquad \text{Equation (A.2)}$$

where $\boldsymbol{u}$ is the velocity, p the pressure, ρ the density, μ the viscosity and k the clot permeability. Outlet boundary conditions are defined through the use of 3-element Windkessel model:

$$Q_{out,ACA} \left(1 + \frac{R_{1,ACA}}{R_{2,ACA}} \right) + C_{ACA} R_{1,ACA} \frac{dQ_{out,ACA}}{dt} = C_{ACA} \frac{dP_{out,ACA}}{dt} + \frac{P_{out,ACA}}{R_{2,ACA}} \qquad \text{Equation (A.3)}$$

$$Q_{out,MCA} \left(1 + \frac{R_{1,MCA}}{R_{2,MCA}} \right) + C_{MCA} R_{1,MCA} \frac{dQ_{out,MCA}}{dt} = C_{MCA} \frac{dP_{out,MCA}}{dt} + \frac{P_{out,MCA}}{R_{2,MCA}} \qquad \text{Equation (A.4)}$$

where Q_{out} is the flowrate at the model outlet, R_1 is the inertial resistance, R_2 is the peripheral resistance, C is the capacitance and P_{out} is the pressure at the outlet. Values for the Windkessel parameters namely C, R_1 and R_2 are given in Table A.1.

Table A.1 Windkessel parameters for each outlet of the ICA bifurcation

Outlet	Symbol	Value	Units	Source
MCA	$R_{T,MCA}$	3.2×10^{9}	Pa·s/m^3	Calculated as $R_T = \frac{P_{mean}}{Q_{mean}} = \frac{60mmHg}{3Q_{ICA}/5}$ using reported pressure[S1-S2] and flow split[36]
	$R_{1,MCA}$	1.79×10^{8}	Pa·s/m^3	Using the relationship[S3] $R_1 = 0.056\ R_T$
	$R_{2,MCA}$	3.02×10^{9}	Pa·s/m^3	Using the relationship[S3] $R_2 = 0.944\ R_T$
	C_{MCA}	4.66×10^{-11}	m^3/Pa	S3
ACA	$R_{T,ACA}$	4.8×10^{9}	Pa·s/m^3	Calculated according to a flow split[36,S4]
	$R_{1,ACA}$	3.33×10^{8}	Pa·s/m^3	Calculated by assuming that the wave speed is the same in all branches.
	$R_{2,ACA}$	4.47×10^{9}	Pa·s/m^3	Difference of 2 resistances ($R_{T,ACA} - R_{1,ACA}$).
	C_{ACA}	2.33×10^{-11}	m^3/Pa	S3

A.2 Estimation of initial concentration of binding sites

A fibrin network is assumed to be composed entirely of fibrin fibres that have an initial length and fibrin fibre density. Fibrin fibres are made up of protofibril strands composed of fibrin monomers. Figure A.1 (a) shows the microstructure of a fibrin fibre.

A fibrin fibre can be discretised into a number of cross-sectional slices that contain binding sites, as shown in Figure A.1 (b). Each asterisk represents protofibrils and spacings between two protofibrils are Δr in the radial direction and Δt in the circumferential direction. By assuming that the cross-sections are circular and distances between each protofibril in both the radial and circumferential directions are constant, the number of protofibrils can be approximated. For the i-th layer, the number of protofibrils can be expressed as:

$$N_{pf,i} = \frac{2\pi}{\vartheta_i} = \frac{\pi}{\sin^{-1}\left(\frac{\Delta t}{2i\cdot\Delta r}\right)} \qquad \text{Equation (A.5)}$$

The total number of protofibrils for a given cross-section can therefore be expressed as:

$$N_{pf,tot} = \sum_{i=1}^{N_L} \frac{\pi}{\sin^{-1}\left(\frac{\Delta t}{2i\cdot\Delta r}\right)} \qquad \text{Equation (A.6)}$$

When the density of fibrin in the clot ρ_0 and the density of fibrin in a fibre ρ_f are known, the ratio between the volume of fibrin fibre to the clot volume can be calculated (ρ_0 / ρ_f = volume of fibre / volume of clot). The total length of fibre per clot volume is therefore expressed as:

$$L_{f,tot} = \frac{\rho_0}{\rho_f} \frac{1}{\pi R_{f0}^2} \qquad \text{Equation (A.7)}$$

and the total number of slices per clot volume is:

$$N_{slice} = \frac{L_{f,tot}}{L_M/2} \qquad \text{Equation (A.8)}$$

where $L_M/2$ is the length of repeating unit in a fibrin fibre (L_M = 45 nm). Yeromonahos et al.[31] reported experimental measurements of average fibre range, interprotofibril distance and number of protofibrils depending on the ionic strength. Based on their experimental data, the values of average fibre radius R_{f0} and interprotofibril distance Δr and Δt are set to be 100 nm and 10 nm, respectively, and the calculated number of protofibrils using Equation (A.8) is 344, which is similar to the measurement (approximately 300 in Yeromonahos et al.[31]). The fibre density ρ_f is chosen to be 0.245 g/mL, based on the values reported by Diamond[30] (0.21 and 0.28 g/mL for fine and coarse clots). Although fine and coarse clots have different interprotofibril distance and fibre density, only the fibre radius R_{f0} is varied in the current simulations to account for changes in permeability and resistance and their effects on clot lysis (R_{f0} being 100 nm and 30 nm for coarse and fine clots, respectively). The density of fibrin in the clot ρ_0 was assumed 3 mg/mL based on fibrinogen levels reported by Undas et al.[S5-S6].

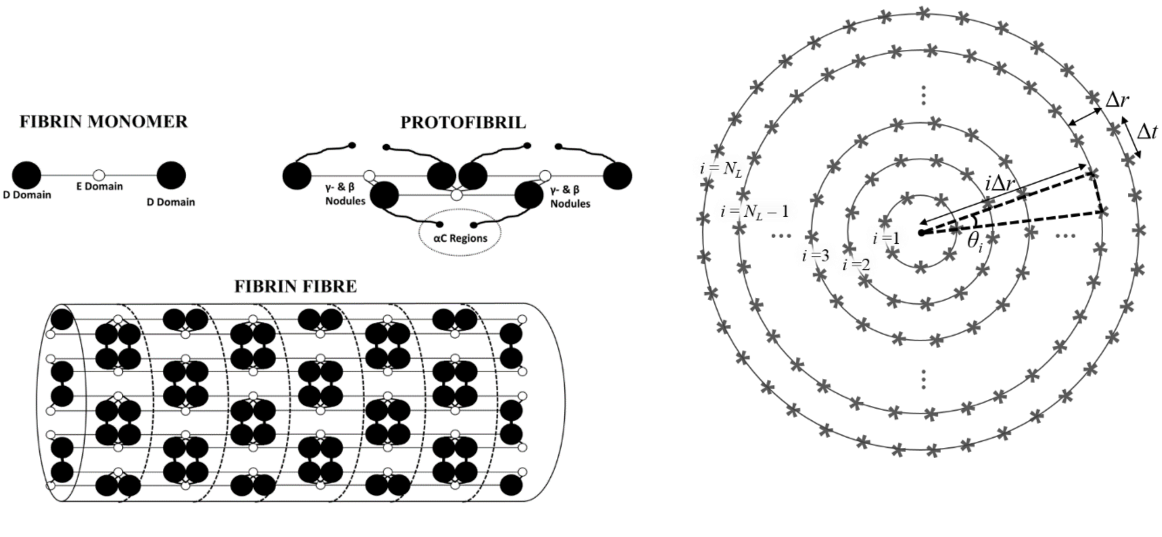

(a) (b)

Figure A.1 Illustration of (a) polymerised fibrins that form a fibrin fibre and (b) binding sites in each cross-section along a fibrin fibre that consists of N_L layers. Δr and Δt are the distance between two protofibrils in the radial and circumferential direction, respectively.

Using the total number of slices and total number of protofibrils per slice, the total number of binding sites per unit volume of clot is:

$$n_{tot} = n_{bs} \frac{N_{slice} N_{pf,tot}}{N_{av}} \qquad \text{Equation (A.9)}$$

where n_{bs} is the number of available binding sites per slice and N_{av} the Avogadro's number and the unit of n_{tot} is the number of moles per unit volume. n_{bs} is assumed to be 2 in this work as Diamond and Anand[27] used a total of 3.5 binding sites per monomer (equivalent to two slices).

Appendix B. Validation of the Compartmental Model

The compartmental model has been validated against three sets of experimental data in the literature[5,18,32]. Although the experiments were carried out on healthy individuals[5] and patients with myocardial infarction[18,32], it is assumed that the pharmacokinetics of tPA and other proteins would remain the same.

Noe and Bell[18] collected blood samples from patients suffering from myocardial infarction who were treated with a continuous alteplase infusion of 0.5 mg/kg over a 60 minute period. The plasma concentrations of PLG and fibrinogen for 13 patients were measured before drug infusion and at 1.5, 2, 4 and 24 hours after treatment. The concentrations of lysis proteins over time for these patients can be found in Figures A.1 (a) and (b) where solid lines show the corresponding predictions by the computational model for an initial concentration of 1.21 µM, 8 µM and 1 µM for PLG, fibrinogen and AP respectively. The activation and generation of PLG show good agreement with the measurements using the model parameters defined in Table 1. Only one parameter, $k_{cat,f}$, required fitting to the measurements. Tanswell et al.[5] evaluated the plasma concentrations of tPA for 12 healthy patients after 30 minute infusion of either 0.25 mg/kg or 0.5 mg/kg alteplase over a 3 hour period. Figure B.2 shows a comparison between the measured and predicted tPA concentration. During infusion, a hepatic clearance coefficient of 0.183 min^{-1} (corresponding to a tPA half-life of just under 4 minutes) has been found to give a good agreement with the experimental data at both tPA concentrations.

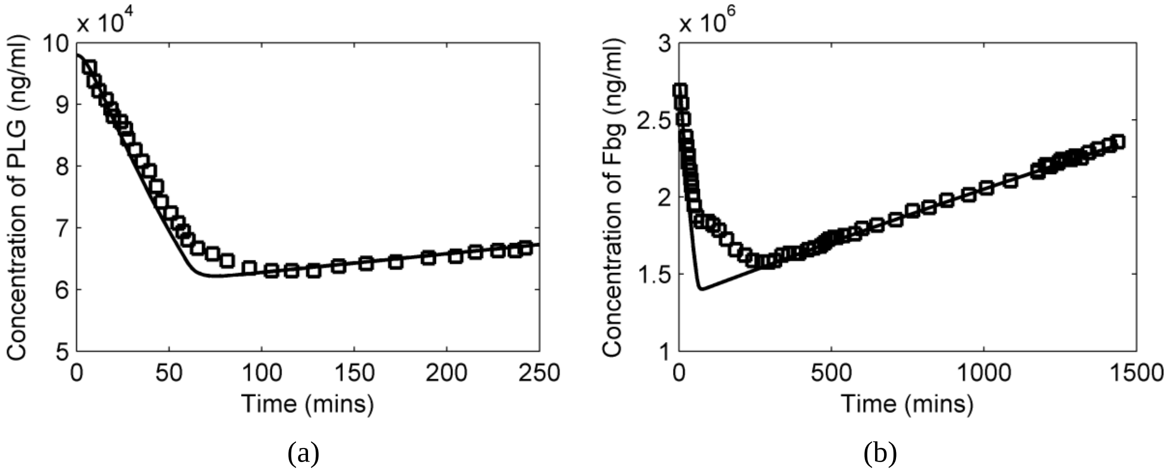

Figure B.1 Comparison of simulation results of the compartmental model (solid line) and experimental data[18] (symbol) for plasma concentrations of (a) PLG and (b) fibrinogen.

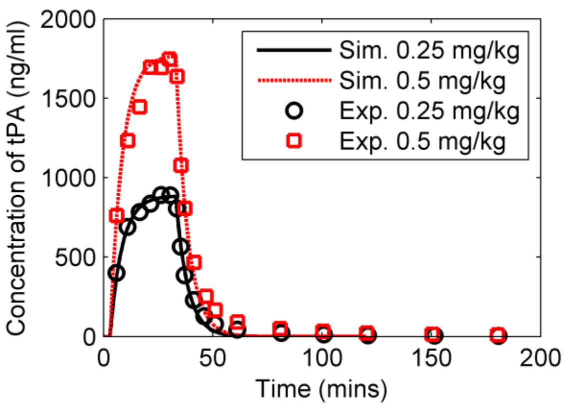

Figure B.2 Comparison of simulation results of the compartmental model (solid line) and experimental data[5] (symbol) for plasma concentrations of tPA.

Collen et al.[32] measured the concentrations of PLG, fibrinogen and AP of 101 patients with acute myocardial infarction who were given a 0.75 mg/kg alteplase infusion over a 90 minute period. Protein concentrations are evaluated with respect to changes in the baseline value at 0, 60 and 90 minutes after infusion as shown for PLG, fibrinogen and AP in Figures B.3 (a), (b) and (c) respectively. The plasma concentrations of PLG, Fbg and AP agree reasonably well with the experimental data using the same Michaelis-Menten coefficients given in Table 1. The plasma tPA concentration predicted by the model under the experimental conditions is shown in Figure A.3 (d) and is consistent with the observation of Collen et al.[32] who found a steady tPA concentration of 1200 ng/ml following infusion.

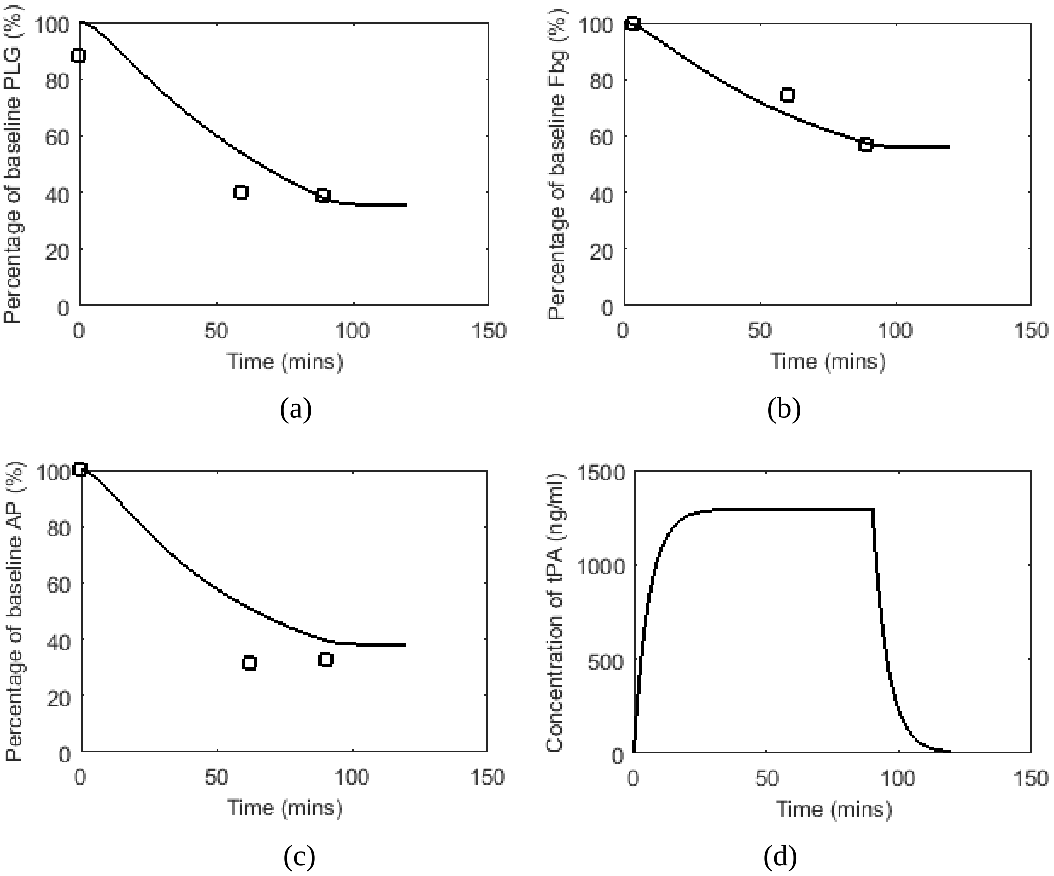

Figure B.3 Comparison of simulation results (solid line) with experimental results[32] (symbol) for plasma concentrations of (a) PLG, (b) fibrinogen, (c) AP and (d) tPA. The initial PLG and AP concentrations are 2 μM and 1 μM in the experiments.

Supporting Information References

S1. Ogoh, S. et al. Middle cerebral artery flow velocity and pulse pressure during dynamic exercise in humans. American Journal of Physiology - Heart and Circulatory Physiology **288**, H1526–H1531 (2005).

S2. Matano, F. et al. Intraoperative middle cerebral artery pressure measurements during superficial temporal artery to middle cerebral artery bypass procedures in patients with cerebral atherosclerotic disease. Journal of Neurosurgery **125**, 1367–1373 (2016).

S3. Xiao, N., Humphrey, J.D. & Figueroa, C.A. Multi-scale computational model of three-dimensional hemodynamics within a deformable full-body arterial network. Journal of Computational Physics **244**, 22–40 (2013).

S4. Grinberg, L., Cheever, E., Anor, T., Madsen, J. R., & Karniadakis, G. E. Modeling blood flow circulation in intracranial arterial networks: a comparative 3D/1D simulation study. Annals of Biomedical Engineering **39**, 297–309 (2011).

S5. Undas, A. et al., Altered fibrin clot structure/function in patients with idiopathic venous thromboembolism and in their relatives. Blood **114**, 4272–4278 (2009).

S6. Undas, A. et al. Fibrin clot properties are altered in patients with chronic obstructive pulmonary disease. Thrombosis and haemostasis **102**, 1176–1182 (2009).
